# The principles of cancer staging

**DOI:** 10.3332/ecancer.2016.ed61

**Published:** 2016-11-24

**Authors:** James Brierley, Mary Gospodarowicz, Brian O’Sullivan

**Affiliations:** Department of Radiation Oncology, University of Toronto, 610 University Avenue, Toronto, ON, M5G 2M9, Canada

**Keywords:** staging, prognosis, disease extent, tumour profile, prognostic factor, cancer control

## Abstract

The anatomic disease extent or tumour stage of a cancer at diagnosis as a determinant of prognosis is discussed. The importance of cancer stage in individual patient prognosis and determination of treatment is reviewed as well as its value in research and cancer control activities. The conflict between the need for stability of cancer stage definitions over time and the need to evolve with advances in medicine are examined. The *e*cancer elearning modules on Cancer Stage are introduced.

## Introduction

The key features of all cancers are the site of the tumour, the tumour profile (which includes histopathology, morphology, molecular, and genetic characteristic of the tumour), and the anatomic disease extent or tumour stage. The latter has been recognized for many years as an important determinant of prognosis for an individual cancer since patients who present with extensive disease almost universally have worse outcomes than those whose disease is much more localized [1, 2]. The classification of anatomic extent of disease, called ’the stage’ is based on the TNM system first developed in Paris in the 1940’s and 1950’s by Pierre Denoix and the Union for International Cancer Control (UICC). In the TNM classification, T category describes the extent of the primary tumour, either by size, depth of invasion or invasion of adjacent structures, the N category indicates the absence or extent of regional lymph nodes metastasis, and the M category indicating the absence or presence of distant metastasis. The combination of TNM categories in a given tumour described at diagnosis before any treatment is applied is called clinical TNM or cTNM. After surgical excision, the pathological TNM or pTNM classification is applied. The cTNM guides the approach to investigation and treatment, while the pTNM guides the use of adjuvant therapies. Both classification give indications of prognosis [3].

## Staging

The activity of ‘cancer staging’ is used to describe the process of determining the anatomic extent of disease. In the past the cTNM assessment relied on clinical examination and the limited imaging available including plain radiographs and ultrasound scanning. For many tumours this was supplemented by exploratory or staging surgery. Significant advances in diagnostic imaging tools such as CT, MRI, ultrasound, and PET imaging have mostly rendered invasive procedures unnecessary. The pTNM is determined from the histopathological findings after surgery sufficient to evaluate the highest T and N categories. Biopsy findings alone are inadequate to assess the pTNM. Once the T, N and M categories have been assigned they are combined into stage groups, the stage group must be recorded and remain unchanged in the medical record.

## The objective of recording cancer stage

The objectives of recording the stage at presentation of a carcinoma have been specified by the UICC (see Table 1) [3]. Physicians involved in the care of patients with cancer understand that cancer staging at diagnosis is a prerequisite to assessing an individual patient’s prognosis and determining the appropriate treatment. They also know, when applying or evaluating evidence based medicine and treatment guidelines or when entering patients into clinical studies, that the stage of the cancer must be known before proceeding. However, the use of recorded stage in cancer control activities is often not fully appreciated by clinicians [4]. Accurate documentation of stage and recording stage in cancer registries allows for evaluation of disease in a population; adding stage to the knowledge of the incidence of a cancer in a particular jurisdiction significantly enhances the value of the data in evaluating cancer burden and facilitating the development of cancer programs for screening and treatment [4]. The needs of a cancer control program are significantly different depending on whether localized or early stage disease or advanced or late stage disease predominates. If the population consists primarily of patients with early stage disease, the need for diagnostic services, surgery and radiation tends to be greater. When the population consists of patients with metastatic disease, investment in palliative and supportive care predominates.

To compare outcomes across jurisdictions and to evaluate the long-term outcomes of populations, stage definitions are needed that are uniform across populations and time [5]. Stability over time is helpful for cancer surveillance but not optimal for clinical use of staging classification. Attempts to minimize changes in staging classification while maintaining is clinical relevance pose significant challenges. There is always a conflict between a classification being used clinically reflecting and supporting the most current treatments and investigations while maintaining stability.

## UICC Cancer Staging Project

Over the years, the UICC has endeavored to make the TNM classifications simple enough for worldwide use but sophisticated enough for academic settings in well-resourced environments, ensuring that changes are evidence-based and, when possible, facilitate comparability over time [6].

While it is widely recognized that the anatomic extent is a powerful prognostic indicator in cancer, many other factors also have a significant impact on determining outcome. These factors can be classified as:

## Disease

- Anatomic Extent of Disease: reflected by TNM.
- Tumour Profile: histopathologic, (i. e., grade) molecular and genetic features of a tumour.

**Patient:** demographic factors such as age and performance status or acquired such as immunodeficiency status.

**Environment:** access to treatment, and healthcare expertise and delivery, socioeconomic factors.

There is a temptation to incorporate other prognostic factors, in particular molecular and genetic tumour characteristics, into anatomic extent of disease. This would confuse the two important axes of classification. The UICC TNM Project recommends restricting the term cancer stage to describe the anatomic disease extent at diagnosis and recommends the use of the term ‘prognostic group’ to describe all factors that guide intervention and affect the outcome. It is important to recognize that tumour profile and the tumour stage are independently useful in clinical and in research settings and therefore they should remain distinct.

## UICC and ecancer

Given the universal importance of cancer stage across all dimensions of the cancer community the UICC and *e*cancer have collaborated on a series of modules for the purpose of educating and informing the global cancer community on cancer stage. There are currently seven modules, that we believe are the only educational modules available on the internet that start with an introduction to cancer staging and the basic knowledge needed to be able to stage patients with cancer and then proceed to the details of individual cancer sites. They are free and approved by the UICC and on completion a certificate is available to download. We recommend that everyone interested in cancer stage starts with the introductory module as it outlines the general principles of the UICC TNM Classification of Malignant Tumours and explains how to apply the principles and rules of staging to all tumour sites and defines additional descriptors and classifications that can be used. There are six site specific modules that describe tumour specific T,N and M category and stage group definitions. The sites covered were chosen to represent common tumours and tumours of global importance to the surveillance community and are: breast cancer, prostate cancer, cancer of the colon and rectum, cervix, lung and lip and oral cavity cancers. Each module takes approximately 30 minutes to complete and includes a voice-over and interactive quiz.

(http://ecancer.org/education/module/161-the-uicc-tnm-classification-system.php)

## Conclusion

Cancer staging is a cornerstone of patient care, cancer research and cancer control activities and as such a basic understanding of the principles behind cancer stage in essential for all involved in all aspects of cancer care. The introductory module and subsequent site specific modules the UICC have produced in collaboration with *e*cancer should be taken by anyone involved in cancer care from individual patient management to the development and implementation of population based cancer control programs.

## Conflicts of interest

The authors declare they have no conflicts of interest

## Figures and Tables

**Table 1.** 

Aims for staging classification are to: aid treatment planning provide an indication of prognosis assist in the evaluation of treatment results facilitate the exchange of information between treatment centres contribute to continuing investigations of human malignancies support cancer control activities
